# Meal replacement and functional connectivity in the brain network for appetite: connecting the dots

**DOI:** 10.3389/fpsyg.2015.00547

**Published:** 2015-04-28

**Authors:** Tanya Zilberter

**Affiliations:** Infotonic ConseilMarseille, France

**Keywords:** meal replacement, appetite, fMRI, obesity, eating behavior

The article by Paolini and colleagues is a part of the Research Topic “The two-way link between eating behavior and brain metabolism” (Zilberter, 2015). The authors were first to report the effect of MR on brain networks during moderate hunger state. They used sophisticated analytical/experimental techniques and observed a new phenomenon, which they reported in a faultless manner. The main message is that a meal replacement (MR) curbs appetite and lowers functional connectivity (FC) in the regions of interest (ROI): insula, anterior cingulate cortex (ACC), superior temporal pole (STP), amygdala and hippocampus.

## Meal replacemdents

### Uses and specifications

MR is a formulation “containing ingredients which are expected to provide nourishment, nutrition, hydration, satisfaction of hunger/thirst, or desire for taste, texture or flavor” as defined by Health Canada (2010). The idea of MRs was first proposed in 1946 when J. L. Gamble from Harvard Medical School suggested the first recipe for a “life raft” survivor's kit (Gamble, 1989). As the era of spaceflight began, MR received much attention from nutritionists and food technologists.

Currently, besides medical formulas for specific health conditions including those requiring parenteral nutrition, MRs are adopted as a convenient tool in the battle against obesity (Hamdy et al., 2008; Wadden et al., 2009) and as such is chosen by the authors. Low compliance is considered to be the biggest obstacle for successful dieting and MR is shown to improve it, especially in the long run (Heymsfield et al., 2003; Wadden et al., 2009; Heymsfield, 2010). It is thought that this strategy works by increasing nutritional awareness, improving meal timing (Wing and Jeffery, 2001) or due to reduced energy content: MR is usually 2–3 times lower in calories than an average meal. Importantly, once a caloric deficit is created hours later, hunger is augmented (Paoli and coauthors demonstrated this as well) but food intake during the next meal is not increased. In the case of skipping breakfasts, for example, it resulted in a negative energy balance of about 400-kcal a day (Levitsky and Pacanowski, 2013).

The authors used a generic, commercial 240-Kcal MR beverage called BOOST, 98% of which consists of corn syrup, sugar, and milk protein. This particular MR successfully “*calmed the hot-state brain network of appetite*.” It should not, however, be taken for granted that this MR is unique in eliciting the reported effect. MRs with increased viscosity (Zijlstra et al., 2008) decreased energy density (Murray et al., 2015) or improved nutrient composition (e.g., low-glycemic, protein-enriched, high in isoflavone, Berg et al., 2008; König et al., 2012) produced more favorable changes in long-term metabolic outcomes, such as improved insulin sensitivity and increased fat oxidation. On the other hand, using a MR containing carbohydrates instead of broadly used artificial sweeteners might indeed have a reward-related advantage: fMRI studies of oral stimulation (mouth rinsing without swallowing) with carbohydrates, both sweet and not sweet (maltodextrin), caused activation in some of the same ROI (e.g., the ACC as in Paolini et al., 2014), while stimulation with a non-carbohydrate sweetener indistinguishable by taste (saccharin) did not (Jeukendrup and Chambers, 2010) thus probably diminishing the hedonic/rewarding properties of non-carbohydrate MRs.

## Obesity

Paolini and coauthors studied FC in older, obese adults and showed that after 2.5 h of food restraint, MR decreased cravings and hunger ratings, which was accompanied by reduced FC in the brain network for appetite (BNA). It is necessary to keep in mind that changes in FC after a MR in obese and non-obese subjects may be different: normal-weight subjects displayed less reduction of FC in the homeostatic, reward and emotion-related brain areas (Frank et al., 2014). Obese women comparing with non-obese exhibited greater fMRI-activation by food-related stimuli in a large number of brain regions involved with motivational effects of food including ACC, insula, hippocampus, and amygdala (Stoeckel et al., 2008). Visceral cues increased fMRI activation in the insula and decreased activation in amygdala among other regions in obese but not normal subjects (Tomasi et al., 2009).

One of comorbidities of obesity is anxiety (Singh, 2014) and subjects with anxiety are routinely excluded from the studies (García-García et al., 2013). Both lower sensitivity to reward and increased FC was shown to coexist in the ROI including the insula (Verdejo-García et al., 2015). Resting-state fMRI of patients with anxiety featured abnormalities in the ROI similar to Paolini and coauthors—in the amygdala, hippocampus, ventral striatum, insula, and ACC (Oathes et al., 2015). Resting-state brain activity is abnormal in anxiety (e.g., Dichter et al., 2015). While screening for schizophrenia, bipolar disorder, MS, Parkinson's, Alzheimer's, dementia, alcoholism, and binge eating, Paolini and coauthors did not exclude anxiety, thus the chance of an influence of this condition on the study outcome remains and should be kept in mind.

## Brain network for appetite

Appetitive behavior is driven by a combined effort of sensory, gastric, and metabolic signals occurring after energy intake, which modulate taste activation in reward areas (Smeets et al., 2011). Paolini et al. emphasized the anatomically central location of the insula in the BNA, on the crossroad between lower-level representations and higher-level processing of visceral cues. Indeed, the insula is activated by gustatory sensing (van Rijn et al., 2015). Its connection with ACC provides the link between gustatory, emotional, cognitive, and behavioral processing (Jeukendrup and Chambers, 2010). PET data from hungry subjects exposed to real food vs. non-food stimuli (Wang et al., 2004) revealed activation in the insula, STP. They also registered activation in orbitofrontal cortex, which Paolini and coauthors did not expect to see due to lack of food cues processing in their protocol.

## Conclusion

**Figure 1:**
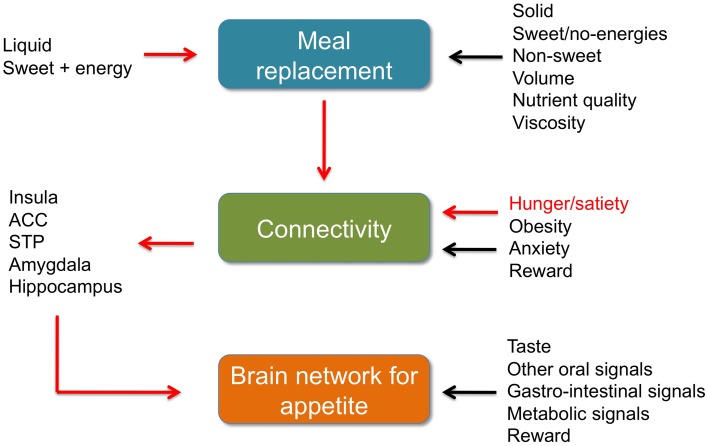
**Meal replacement, functional connectivity and BNA**. Brief summaries. Red arrows: data by Paolini et al. (2014). Black arrows other data discussed in the commentary, references in the text. ACC, anterior cingulate cortex. STP, superior temporal pole.

The accurate finding of Paolini and coauthors concern the vulnerable, growing population of overweigh/obese, aging men and women, free from neurological and neurodegenerative disorders and binge eating, not screened for the anxiety disorder. MR (liquid, carbohydrate-based, low-viscosity and low-fiber, 240-Kcal) calmed the “hot-state brain network of appetite”, which was activated by 2.5 h of food deprivation. It is important to further investigate whether or not the effect would be observed on young, lean, anxiety-free subjects, with this or other types of MR. The findings of Dr. Brielle Paolini and colleagues are thought-provoking and will definitely find a well-deserved place in the bigger picture of the appetite regulation mechanisms (Figure 1).

### Conflict of interest statement

The authors declare that the research was conducted in the absence of any commercial or financial relationships that could be construed as a potential conflict of interest.
